# Enabling Context-Aware Data Analytics in Smart Environments: An Open Source Reference Implementation

**DOI:** 10.3390/s21217095

**Published:** 2021-10-26

**Authors:** Andres Munoz-Arcentales, Sonsoles López-Pernas, Javier Conde, Álvaro Alonso, Joaquín Salvachúa, Juan José Hierro

**Affiliations:** 1 Departamento de Ingeniería de Sistemas Telemáticos, ETSI Telecomunicación, Universidad Politécnica de Madrid, 28040 Madrid, Spain; javier.conde.diaz@upm.es (J.C.); alvaro.alonso@upm.es (Á.A.); joaquin.salvachua@upm.es (J.S.); 2 Departamento de Sistemas Informáticos, ETSI Sistemas Informáticos, Universidad Politécnica de Madrid, 28031 Madrid, Spain; sonsoles.lopez.pernas@upm.es; 3 FIWARE Foundation, 10587 Berlin, Germany; juanjose.hierro@fiware.org

**Keywords:** context-aware systems, big data, data analytics, smart environments, IoT, machine learning, FIWARE

## Abstract

In recent years, many proposals of context-aware systems applied to IoT-based smart environments have been presented in the literature. Most previous works provide a generic high-level structure of how a context-aware system can be operationalized, but do not offer clues on how to implement it. On the other hand, there are many implementations of context-aware systems applied to specific IoT-based smart environments that are context-specific: it is not clear how they can be extended to other use cases. In this article, we aim to provide an open-source reference implementation for providing context-aware data analytics capabilities to IoT-based smart environments. We rely on the building blocks of the FIWARE ecosystem and the NGSI data standard, providing an agnostic end-to-end solution that considers the complete data lifecycle, covering from data acquisition and modeling, to data reasoning and dissemination. In other words, our reference implementation can be readily operationalized in any IoT-based smart environment regardless of its field of application, providing a context-aware solution that is not context-specific. Furthermore, we provide two example use cases that showcase how our reference implementation can be used in a variety of fields.

## 1. Introduction

Context can be defined as “any information that can be used to characterize the situation of an entity, where an entity can be a person, place, or object that is considered relevant to the interaction between a user and an application, including the user and applications themselves” [1]. As such, context-aware systems are those that can use context information for performing intelligent or smart actions [2]. According to [3], one of the goals of context-aware systems is to capture and process the information in the context of a device to provide services built on a particular person, place, time, event, etc. Worded differently, context-aware systems capture context, analyze and interpret the context, and make adjustments to the system’s behavior for the user’s changing situation without explicit intervention from the user.

Currently, one of the most promising application fields of context-aware systems is that of IoT-based (Internet of Things) smart environments. A smart environment is a physical space that relies on technology to connect “things” to the virtual world, increasing the level of awareness of what is occurring in physical environments. In addition, IoT devices and IoT-based smart environments include software platforms and services, artificial intelligence (AI) and machine learning (ML) capabilities, big data processing, cloud computing, heterogeneous connectivity, virtual/mixed realities, and a huge range of technologies to improve people’s quality of life, to decrease environmental impact, and to optimize the use of physical resources [4]. According to [5], IoT-based smart environments can be classified in the following areas: smart cities, smart homes, smart grid, smart buildings, smart transportation, smart health, and smart industry. Many proposals of context-aware systems applied to these different IoT-based smart environments have been presented in the literature [6,7,8,9,10].

Due to the increasing availability of devices that collect data on a continuous basis, the development of context-aware systems is closely related to that of big data systems, especially IoT-based smart environments. As such, the 5Vs of big data need to be considered: volume, velocity, variety, veracity, and value. The overwhelming number of devices (volume) that are constantly deployed for context data collection beg the need for solutions like distributed storage to be able to persist the data for post-hoc analysis. Moreover, the fluctuating nature of some of the variables measured by context-related devices (e.g., temperature, pollution, presence, etc.) make it necessary to handle the data in real time and thus deploy technologies capable of handling such a high velocity of data. Regarding variety, one of the key aspects of context-aware systems is that they may include data from multiple sources and, at the same time, a single data source may be used for multiple applications. Therefore, such systems need to separate the process of how the context is captured from how it is used, as pointed out by [2]. By so doing, applications may use contextual information without accounting for the specific details of devices or how they have been implemented. Moreover, when it comes to veracity, context-aware systems need to consider the security and privacy aspects of data, providing the right balance between privacy and system potential. Lastly, the value of data are related to the potential social or economic value that the data might create, i.e., the possible applications of the data collected.

A number of prior works have provided frameworks and architectures for designing context-aware systems. The majority of existing works often provide a generic high-level structure of how a context-aware system can be operationalized, but do not offer many clues on how to implement it. On the other hand, there are many implementations of context-aware systems applied to specific IoT-based smart environments use cases (e.g., smart farming, smart cities). However, although these implementations are context-aware, they are also context-specific: it is not clear how they can be extended to other use cases. Moreover, although recent literature on context-aware systems increasingly addresses the challenges brought by the 5Vs of big data, due attention to the issue of data standardization or harmonization is yet to be paid. This issue is critical in collaborative scopes, where several systems exchange relevant contextual information based on detected complex events.

In this article, we aim to provide a reference implementation that can be used in any context-aware system development of an IoT-based smart environment. For that purpose, we rely on the building blocks of the FIWARE ecosystem and the NGSI data standard, providing an agnostic end-to-end solution that takes into consideration the complete data lifecycle as well as the issues derived from big data requirements, filling the existing gap in the literature. In other words, our reference implementation can be readily operationalized in any IoT-based smart environment regardless of its field of application, providing a context-aware solution that is not context-specific. We provide two use cases that showcase how our reference implementation can be used in a variety of fields, covering from data acquisition and modeling, to data reasoning and dissemination.

The remainder of the article is structured as follows. The next section presents related work on context-aware systems and their specific application to IoT-based smart environments. In Section 3, the data standardization process is described. Section 4 presents an overview of the conceptual representation of the architecture and the description of each of its layers. Section 5 shows the implementation of the previous architecture using FIWARE GE’s including the data modeling and the building blocks. In Section 6, two use cases are presented in two different application scenarios in which our implementation has been operationalized. Lastly, Section 7 presents the conclusions of the article and proposes some lines of future work.

## 2. Related Work

### 2.1. Context-Aware System Architectures

Researchers have diverging opinions when it comes to how to structure a context-aware system. In the work by [11], the authors presented a conceptual framework for context-aware systems segmented into five layers: sensors, raw data retrieval, preprocessing, storage and management, and application. Not long after, the authors of [3] presented an abstract architecture for context-aware systems based on a thorough review of the literature, in which four layers were included: network, middleware, application, and user infrastructure. Although the latter proposal shows a more generalizable way of representing context-aware systems, both of them fail to cover the integration of new devices like IoT and to take into consideration the security aspects. A more recent study by [12] presented a context-aware middleware cloud approach for integrating precision farming facilities into the IoT toward agriculture 4.0. This proposal also presented the conceptual architecture of context-aware systems divided into three layers: physical layer, middleware layer, and application layer. Although this last proposal shows a higher level of abstraction of the conceptual model, it was contextualized in the field of Precision Farming and its operationalization was limited only to that scenario.

Although the number of layers in which context-aware architectures are segmented differs across the literature, most of them share the same key elements combined in different configurations. For example, in the works mentioned above, the sensors and raw data retrieval layers proposed by [11] are equivalent to the network layer proposed by [3] and to the physical layer described in [12]. Regardless of how the different elements in the architecture are organized, a crucial aspect to take into account is data standardization, which provides an effective communication mechanism between the different layers and also with the outside or complementary systems.

### 2.2. Implementations of Context-Aware Systems to IoT-Based Smart Environments

A burgeoning number of implementations of context-aware IoT-based smart environments have been developed in the last few decades. In the case of Smart Transportation, proposals like a taxi-aware map [13] present the development of context-aware systems for identifying and predicting vacant taxis in the city, based on three parameters: time of the day, day, and weather conditions. These systems use contextual information provided by a historical record of data stored in a database, for building an inference engine, using a naïve Bayesian classifier to make the predictions. For building the predictor, a dataset with GPS traces of 150 taxis in Lisbon, Portugal was used. As a result, they provide a system capable of predicting the number of vacant taxis in a 1 × 1 km^2^ area with a 0.8% error rate. Additionally, the authors of [14] present a platform designed to automate the process of collecting and aggregating context information at a large scale. They integrate services for collecting context data like location, users’ profile, and environment, and validate that platform through the implementation of an intelligent transportation system to assist users and city officials to better understand traffic problems in large cities. They use domain-specific ontologies to describe events, dates, locations, user activities, and relations with other people and objects. In addition, a set of XML-based format rules are defined for triggering a series of actions when certain conditions are met. The most recent work was provided in [15]. In this article, a recommendation system that offers multi-modal transportation planning which is adaptive to various situational contexts is presented. They use multi-source urban context data as an input to define two recommendation models using gradient boosting decision tree and deep learning algorithms for building multi-modal and uni-modal transportation routes. They conclude that their extensive evaluations on real-world datasets validate the effectiveness and efficiency of that proposal.

Although the previous works present suitable proposals of context-aware systems in the field of smart transportation, they also provide some insights into the challenges that need to be addressed. Scalability is one of the most relevant concerns expressed in those articles. The need to provide ways not only to capture context but also to process it efficiently must be considered. Another important challenge they identify is the need for unifying the way to capture and store the data; the presented proposal uses its methods and structure for dealing with this topic; as a result, many compatibility issues can be derived from this in the case that many systems need to share data or coordinate between them.

Moreover, context-aware systems have been operationalized in the development of smart homes and smart buildings. The authors of [16] presented a context-aware wireless sensors system for IoT-centric energy-efficient campuses. They used context-based reasoning models for defining transition rules and triggering to reduce the energy consumption on a university campus. Another study [17] described a proposal for creating an elevator system in smart buildings capable of reducing the passenger waiting time by preregistering elevator calls using context information. This system captures the context data of the location of the passenger and, based on a 3D localization model, makes automatic preregistration to the elevator considering the distance between the passenger and the elevator. These two proposals are only two examples of how context-aware systems play a key role in any space considered smart. However, these two proposals present some flaws in terms of how heterogeneous and unstructured data are managed.

One of the most widespread uses of context-aware systems is in Smart Industry, specifically in Smart Farming. In [18], the authors present a context-aware platform serving as a middleware mechanism for interfacing environmental sensors with IoT and Cloud for providing real-time process control agriculture application. To achieve that goal, they combine many software platforms and tools, such as IBM Bluemix IoT for managing the IoT data, a Java application for data acquisition and storing in the cloud, and a C# application for handling stored data. One of the most novel approaches was presented in [12], in which a complete framework is proposed including a middleware service that acts as a decision support system for the entire framework. Moreover, several reviews of the IoT technologies used in agriculture have been performed in the last few years [19,20,21,22]. These reviews concluded that, despite the fact that IoT technologies are continuously advancing and promoting the creation of novel agricultural applications and services, some critical issues concerning the interoperability as well as the semantic annotation of heterogeneous data need to be addressed. To that end, in recent years, many efforts have been developed not only for providing data semantics and interoperability within this type of environment but also for including the data models, unified standards, vocabularies, connectors, and platforms. Many of these efforts have been materialized as different proposals for different application scenarios [23,24,25,26,27,28]. All of these works have one thing in common: they consider the use of FIWARE as an enabler open-source platform for providing context management, storage, data analytics, and security for each of their services. A summary of the discussed context-aware applications in smart environments is provided in Table 1.

According to [29], a wide range of studies carried out on the topic of context-aware systems and big data are limited to case studies without the use of any common conceptual frameworks. As such, little attention has been paid to the integration of context-aware computing and big data analytics in the field of smart environments. In the same article, the authors conduct a review of the literature including architectures for pervasive context-aware services in smart spaces in terms of middleware components and prototype applications, middleware for context representation and management in pervasive computing, middleware-based development of context-aware applications with reusable components, middleware for real-time systems, and so forth. They concluded that there is a need for further research in the area of middleware concerning the use of large-scale context-aware applications, as well as the modeling and management of context information in distributed pervasive applications in open and dynamic pervasive environments. They also determine that it could be advantageous to pursue open standards for designing and implementing solutions for various domains, since such applications involve large-scale and heterogeneous data systems. On the other hand, after an exhaustive literature survey, the authors of [30] identify that only a few of the published articles relate context-aware computing to big data. Thus, based on those findings, they present a set of key challenges that need to be addressed if existing small scale context-aware applications want to be adapted to large scale scenarios. These challenges are related to data heterogeneity, unreliable or redundant data or loss of data, local vs. centralized processing, efficient communication infrastructures, scalable context inference algorithms, and collaborative knowledge transfer of big data. Recent studies have set out to address some of the aforementioned issues [31,32] such as collecting and merging data from heterogeneous sources, using message queues and brokers to facilitate agent communication, and providing a context event processing of data to provide personalized context-aware services. However, proposals like the presented still fail to provide an architecture for collaborative scopes, where several systems can exchange relevant contextual information based on detected complex events, due to the lack of data standardization or harmonization process for exchanging the data. Therefore, the architecture proposed in this article fills the gap found in the existing literature by providing a full context-aware system based on big data technologies, allowing for defining a common framework capable of offering a standardized way to manage context data with a common semantic model and data structure predefined for each application domain (agriculture, health, airports, etc). In terms of scalability, our proposal provides a fully distributed service-oriented model, allowing for increasing and reducing resources according to the demand at each stage of the system. For example, the brokers for managing the data sources are designed to scale and also the processing engines which can parallelize the tasks according to the system’s needs. Furthermore, most of the implementations available in the existing literature are not shown in detail. In this regard, our proposal provides a full description of every component of the architecture at the implementation level, providing a set of software components that can be used without the need for developing them from scratch, but rather focusing on configuration and integration aspects. This is achieved thanks to the fact that all of the components of the architecture are publicly available as open-source software. Lastly, our architecture is fully oriented to collaborative scopes, allowing for exchanging and sharing all the contextual information gathered and generated by the systems that compose the smart space.

## 3. Data Standardization

When modeling data, there are two main alternatives: (1) developing a new data model for each context entity, or (2) modeling the data using data models widely extended in the industry. The second option is preferable because it eases the integration with external systems, and it is less error-prone as these data models are, in most cases, developed by standards organizations and validated by the developers community. In this section, we propose the NGSI-LD standard for modeling the data in our reference implementation. We further propose using available smart data models to ease interoperability with other systems existing in the industry.

### 3.1. NGSI-LD

The Next Generation Service Interfaces-Linked Data (NGSI-LD) API (Application Programming Interfaces) is an evolution of the NGSI interface (NGSIv2) for managing context information following the principles of linked data. It was standardized by ETSI (European Telecommunications Standards Institute) with the aim of integrating the NGSI entities in the semantic web. The API defines a set of HTTP methods for creating, reading, updating, and deleting entities.

In NGSIv2, context information is modeled as a set of entities (i.e., a representation of a real object). An entity is determined generically by its type (e.g., Building) and specifically by its identifier (e.g., Building:1). The state of the entity is modeled with attributes which include the name of the attribute (e.g., height), the type (e.g., Integer), and the value (e.g., 30). Lastly, the attributes can be enhanced with metadata, including again its name (e.g., unitCode), type (e.g., String), and value (e.g., FOOT). Consequently, it resulted in a rigid model, difficult to integrate with external systems with their own definition of each entity (e.g., Building2). In contrast, NGSI-LD was developed to increase interoperability and enable building a graph of knowledge through the establishment of relationships. In the NGSI-LD case, the center piece of context information is again the entity, which is defined by its type (e.g., Building) and its identifier (e.g., urn:ngsi-ld:Building1). The difference from NGSIv2 is that both the identifier and the entity type are identified unequivocally by URIs. In NGSI-LD, there are not attributes: instead, there are properties (e.g., height with value 30) and relationships (e.g., hasParking with value urn:ngsi-ld:Parking1), defined with URIs too. In the case of NGSI-LD, there are no metadata for properties, but instead there can be properties of properties, relationships of properties, properties of relationships, and relationships of relationships. Based on a set of entities, along with their properties and relationships, a Resource Description Framework (RDF) graph can be established composed by triples in the form of:

<subject (URI or blank node)> <predicate (URI)> <object (URI, literal, or blank node)>

                                    with the following possible structures:

                                         <entity> <property> <value>;

                                         <entity> <relationship> <entity>;

                                    and the mentioned variants:

                                         <property> <property> <value>;

                                         <property> <relationship> <entity>;

                                         <relationship> <property> <value>;

                                         <relationship> <relationship> <entity>.

### 3.2. Smart Data Models

The FIWARE Smart Data models (FIWARE Smart Data models: https://smartdatamodels.org, accessed on 9 May 2021) is an initiative that aims to integrate reference data models with the NGSI-LD API. As mentioned above, the NGSI-LD API is agnostic to its domain of application. In other words, it provides mechanisms to access context information, but it does not define how the context information is modeled. This initiative is promoted by the FIWARE Foundation (The FIWARE Foundation: https://www.fiware.org, accessed on 9 May 2021) in collaboration with other institutions as TM Forum (TM Forum: https://tmforum.org, accessed on 9 May 2021), IUDX (India Urban Data Exchange: https://iudx.org.in, accessed on 9 May 2021), or OASC (Open & Agile Smart Cities: https://oascities.org, accessed on 9 May 2021). All the data models are public and they grant the adopters the rights to freely use, modify, and share the modifications. Multiple institutions are participating in this initiative both by publishing new data models (e.g., Atos (Atos: https://atos.net, accessed on 9 May 2021), Telefónica (Telefónica: https://www.telefonica.com, accessed on 9 May 2021), Universidad Politécnica de Madrid (Universidad Politécnica de Madrid: https://www.upm.es, accessed on 9 May 2021)), and by validating them through their adoption in industry and research projects (e.g., KWR Water Research Institue (KWR Water Research Institue: https://www.kwrwater.nl, accessed on 9 May 2021), Métropole Nice Côte d’Azur (Métropole Nice Côte d’Azur: https://www.nicecotedazur.org, accessed on 9 May 2021), National Technical University of Athens (National Technical University of Athens: https://www.ntua.gr, accessed on 9 May 2021)). Smart data models are compliant with both NGSIv2 and NGSI-LD, coded in JSON and JSON-LD, respectively.

At this time, more than 550 data models have been defined spread over more than 11 domains (e.g., Smart Cities, Smart Energy, Smart AgriFood, Smart Aeronautics). Details of all data models are published on GitHub (Smart Data Models GitHub organization: https://github.com/smart-data-models, accessed on 9 May 2021).

## 4. Architecture

**Figure 1:**
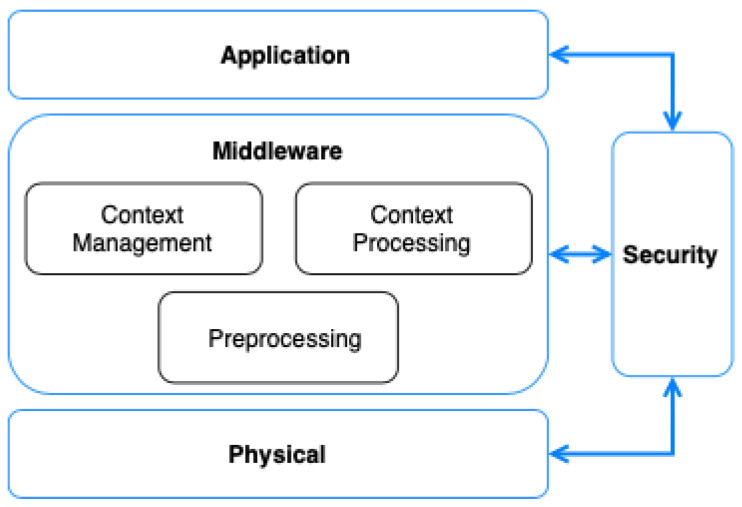
Abstract architecture for context-aware systems.

As discussed in Section 2, context-aware systems usually present an architecture divided into several layers. Depending on the field of application, these layers can be expanded or redistributed. However, in the case of smart environments, a wide range of data sources and systems are involved. One of the problems that are currently presented in these types of ecosystems is the need for managing the vast amount of data that is continuously generated. Thus, in this paper, we consider that a fully compatible architecture for managing context-aware systems in a smart environment not only needs to include context managing but also the necessary big data components for processing the context information. As such, we propose a four-layer architecture that covers the whole context data life-cycle in smart environments. Figure 1 shows the general architecture proposed, which is divided into physical, middleware, application, and security layers.

### 4.1. Physical Layer

Data sources in smart environments are heterogeneous by nature. Thus, accounting for this characteristic is a central part of the architecture of smart environments. The physical layer in our architecture provides all the tasks related to collecting data from heterogeneous sources. On the one hand, this layer includes common IoT, wired and wireless sensors and actuators that often use well-defined communication interfaces and protocols like MQTT, COAP, OMA-LWM2M, OneM2M, etc. On the other hand, systems that generate data in a structured and unstructured way are also included in this layer. Examples of such systems are log servers, web applications, REST services, information systems, and databases. These sources of information have another particularity: they share data using multiple communication protocols. For example, data can be shared through transport messages via TCP/UDP or retrieved by querying a database. Collecting data is not the only process that needs to be performed over the data sources. However, the physical layer is only in charge of providing communication mechanisms for capturing the data. In turn, all the aspects of data transformation, standardization, and cleaning are performed in the middleware layer.

### 4.2. Middleware Layer

The middleware layer provides all the operations needed for managing the complete context life-cycle after its collection. First, the raw data coming from the physical layer need to be preprocessed so it can be accessed by the rest of the components of the architecture in a unified way as well as further processed if complex operations on the data are needed.

#### 4.2.1. Preprocessing

Data coming from the physical layer can be real-time data (streaming) or static data (batch). The way in which each of these data types is handled is different. In the case of streaming data, accounting for its instability is crucial. Moreover, this type of data is usually structured in small chunks. Therefore, raw data can be deleted after preprocessing, without the need to store the complete original dataset. In the case of batch data processing, the regular ETL (Extract Transform and Load) procedure can be applied. For example, when retrieving data from a database, the first step is to define the extraction mechanism (polling, programmed, incremental, etc.). Then, a data filtering and cleaning process for discarding duplicates, redundant, and erroneous data should be applied. Finally, the data must be sent to the correspondent recipient system. Regardless of the nature of the data, it is necessary to provide a way to facilitate the definition and capture of the context. This can be achieved through standardization of the data to a common format and structure. Some of the components that facilitate this process are known as data-flow management systems, one specific implementation of those is Apache NiFi (Apache NiFi: https://nifi.apache.org, accessed on 22 July 2021), which is a software capable of capturing and processing raw data from streaming, batch, and heterogeneous data sources, as well as of transforming this data to the NGSI-LD format through different processors. At this point, data are ready to be used by the next component of this layer: the Context Management.

#### 4.2.2. Context Management

According to [9], context life-cycle refers to how data are gathered, modeled, processed, and how knowledge is deduced from the captured data. In this regard, the context management component is where the logic for representing the context is defined. There are many techniques available for data modeling, e.g., logic-based, markup, key-value, object-oriented, graphical, and ontology-based [33]. In our proposal, the ontology-based modeling technique was selected, taking advantage of the wide set of smart data models currently available. These data models allow defining multiple entities and attributes with a common structure for multiple application domains as was stated in Section 3.2. The context management component is also responsible for providing an interface to access the modeled context data, acting as a context broker between the physical layer and the application layer through a publish/subscribe mechanism. This system allows for retrieving the context information previously modeled using two different approaches, querying and subscribing. Querying allows for accessiing the current context information available. In turn, subscribing allows for receiving notifications when context information changes. Ontology-based reasoning capabilities are introduced in this component, which allow for defining rules to notify users of context changes only when some special behavior is reached. For example, a system can subscribe to changes in Entity “A” if the attribute *b* is greater than 10. If that condition is not met, a notification will not be sent even if the value of *b* changes in any other way.

Context storage is also an aspect to consider in context management. The context broker only stores the latest context information available. Thus, another component needs to provide connections to multiple storage systems, capable of storing the context data in relational and non-relational, graph, cloud-oriented, or distributed databases. By doing so, it is possible to keep track of the evolution of context data. It is important to remark that data standardization is also applicable to the storage of context data. This process is also achieved through the NGSI-LD standard that defines guidelines like name conventions, for tables databases, tables, and column attributes mapping some encoding parameters for saving context data in different storage systems. Although context management provides some basic reasoning functionalities using context, in a wide range of applications, more complex tasks need to be performed, e.g, complex event processing and machine learning. As such, in this proposal, the component of context processing is introduced for extending the features that context management provides.

#### 4.2.3. Context Processing

The context processing component is introduced to handle the processing of large amounts of context data coming from smart environments. As was stated before, smart environments generate high volumes of data with high velocity. Thus, traditional methods for data processing in which this process is done by a single computing instance is not sufficient. Instead, it is necessary to distribute or parallelize the processing jobs. Hence, in the context processing component of our proposal, the processing capabilities rely on well known big data processing engines. A processing engine is a software system in charge of performing operations on data. It usually consists of a series of computing nodes that implement some clustering technology for parallelizing operations in order to process data at a large scale. Some of the most well-known technologies for this purpose are Apache Spark (Apache Spark: https://spark.apache.org, accessed on 22 July 2021) and Apache Flink (Apache Flink: https://flink.apache.org, accessed on 22 July 2021). These technologies offer a programming interface that facilitates the task of performing transformations on data. Both of them provide a wide assortment of resources and libraries for processing data both in streaming and batch modes, such as complex event processing and machine learning. For example, Spark offers the MLLib library which provides a set of resources and algorithms that can be used for building, e.g., recommendation systems, predictors, classification systems, deep learning, and more.

### 4.3. Application Layer

The application layer includes the components that provide high-level services. It serves as an interface between the user and the middleware layer to provide different solutions that need to be developed for each application field. These can be provided as generic Software as a Services (SaaS) solutions or a specific development can be created ad hoc. From a functional point of view, some examples of services included in the application layer could be dashboard views, artificial intelligence systems, advanced analytic, real-time monitoring, data sharing, etc. The data that need to operationalize these systems can be retrieved from the middleware layer, either directly through the context management component or through the context processing component if complex and costly transformations are necessary before the data can be consumed by the applications.

### 4.4. Security Layer

Ensuring security and privacy is essential in any context-aware system due to the own nature of the data generated and collected [34]. This becomes more important when such systems collect information from smart environments like Smart Health, where security and privacy play a key role. In this proposal, a security layer has been included for securing the data exchange between all of the components of the architecture. The security aspects of authentication and authorization are provided by an access control mechanism based on the XACML reference architecture [35]. We consider a combination of Attribute-Based Access Control (ABAC) and Role-Based Access Control (RBAC) [36,37,38] to control the access of users, groups, or applications to individual contextual data based on their level of privacy. Moreover, using authentication and authorization models that allow for defining access policies in the scope of different scenarios or applications make their definition more flexible. This is possible thanks to protocols like OAuth 2.0 [39].

## 5. Implementation Using FIWARE

**Figure 2:**
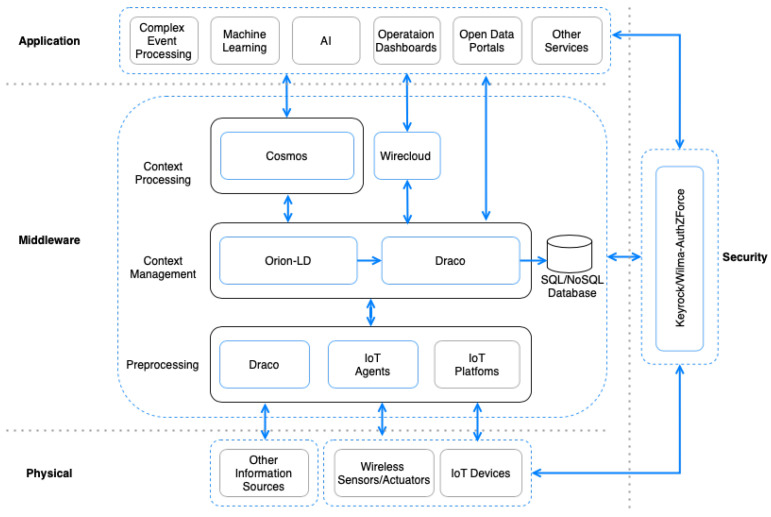
Reference Implementation using FIWARE GEs.

In this section, we present the complete reference implementation based on the architecture detailed in Section 4 (Figure 2). Our implementation relies on the building blocks of FIWARE, which are called Generic Enablers (GEs). Each GE is responsible for providing a specific feature that is necessary for handling data in smart environments. They can be easily configured for a specific setting without the need to develop ad-hoc software components. Our reference implementation relies on the FIWARE GE for the implementation of each of the components of the architecture. Moreover, the NGSI standard, described in Section 3, is the official format supported by all the GEs, easing communication among each one of them.

### 5.1. Physical Layer

In this section, we describe the GEs that are used for providing an interface to interact with IoT devices, wireless sensor networks, and other third party systems.

- **IoT Agent** (FIWARE IoT Agents: https://github.com/FIWARE/catalogue/blob/master/iot-agents/README.md, accessed on 22 July 2021)—It is a set of software modules handling South IoT Specific protocols and the North OMA NGSI interaction. These agents allow for working with the IoT devices that use communication protocols like LWM2M over CoaP, JSON, or UltraLight over HTTP/MQTT, OPC-UA, Sigfox, or LoRaWAN. IoT agent abstracts the communication protocols used by devices by translating them to the FIWARE NGSI format. By using this component, the data collected by IoT devices can be sent to the Context Broker, where each device is represented as an NGSI entity. Additionally, the IoT agents GE allows for triggering commands to actuation devices just by updating specific command-related attributes in their NGSI representation in the Context Broker.

Another set of incubating GEs is provided for managing other devices or systems that are not considered by IoT Agents. This component allows for interacting with a wide range of devices and systems providing complete compatibility with smart environments. The following list presents some of these GEs and their description:

- **Fast DDS** (Data Distribution Service) (FIWARE Fast DDS (Data Distribution Service): https://github.com/eProsima/Fast-RTPS, accessed on 22 July 2021)—It is an incubated GE that has been adopted as the default middleware in ROS2 (Robot Operating System: https://docs.ros.org/en/foxy, accessed on 22 July 2021), the widely known Robot Operating System. Therefore, it helps to interface with ROS2-based robotics systems.
- **OpenMTC** (OpenMTC: https://fiware-openmtc.readthedocs.io, accessed on 22 July 2021)—The OpenMTC Incubated GE brings an open-source implementation of the OneM2M standard.
- **Micro-XRCE-DDS** (Micro-XRCE-DDS: https://github.com/eProsima/Micro-XRCE-DDS, accessed on 22 July 2021)—It is a GE that provides a lite version of the DDS middleware, adapted to run in extremely constrained resource devices (e.g., micro-controllers).

### 5.2. Middleware Layer

In this section, we describe the GEs that take care of the data operations performed in the Middleware layer.

#### 5.2.1. Context Management

Context Management is the central piece of the whole architecture. It handles the whole context lifecycle, providing a standard way to manage, store, and model the context. As stated in Section 4.2.2, a piece of software that provides these capabilities is known as a Context Broker. In this regard, FIWARE has a set of GEs that provide context management capabilities using NGSI-LD as standard. The following is a list of the implementations currently available: Orion-LD Context Broker, Scorpio Broker, and Stellio Context Broker. Our reference implementation is based on the Orion-LD Context Broker, which is the most extended GE and the one endorsed by the European Commission as a Connecting Europe Facility (CEF) Building Block [40].

- **Orion-LD** (FIWARE Orion: https://fiware-orion.readthedocs.io, accessed on 22 July 2021): The Context Broker (Orion-LD) GE manages the entire lifecycle of context information including updates, queries, registrations, and subscriptions. It manages context information through the implementation of a publish–subscribe system through an NGSI interface. Users can create context elements, query and update them, and subscribe to changes in context information that they can receive as notifications. Other elements interact with Orion through HTTP/HTTPS requests. The Context Broker offers the FIWARE NGSI-LD and NGSIv2 (Next Generation Service Interface) APIs and associated information model (entity, attribute, metadata) as the main interface for managing context data [41].

In this architecture, Orion-LD provides a complete solution for managing the latest context data available. However, storing the evolution of context data needs to be performed separately. For that purpose, our reference implementation relies on the Draco GE, which receives each context update as a stream of data and injects them into multiple data storage systems:

- **Draco** (FIWARE Draco: https://fiware-draco.readthedocs.io, accessed on 22 July 2021) is a dataflow management system based on Apache NiFi that supports powerful and scalable directed graphs of data routing, transformation, and system mediation logic using a set of processors and controllers. Draco is aimed at providing storage of historical context data, allowing for receiving data events and dynamically recording them with a predefined structure in several data storage systems. In the scope of this implementation, Draco is proposed to cover the historical context storage in the Context Management component.

#### 5.2.2. Preprocessing

As stated in Section 4.2.2, in this proposal, we also consider alternative data sources that can feed the context management and processing components. On the one hand, there are REST APIs or Database systems that need to be queried periodically. On the other hand, there are systems like TCP or HTTP servers that send a continuous flow of data. These data, possibly coming from external applications, also need to be converted into NGSI-LD data and sent to the Context Broker. In order to deal with these types of systems that are not covered by the IoT Agents, FIWARE relies on the **Draco GE**. The suite of processors and controllers provided in this GE allows for converting the incoming data into NGSI-LD entities and attributes, required for their publication in the Context Broker.

#### 5.2.3. Context Processing

The context processing component relies on big data technologies to extract valuable insights or derive smart actions. Although big data frameworks provide well-defined interfaces and systems that can be integrated with a wide range of systems and devices, standardized data injection is still an open issue in these systems. In this regard, FIWARE provides a set of connectors and libraries that allow for processing NGSI (NGSIv2 and NGSI-LD) context data using two of the most well-known big data frameworks through the Cosmos GE.

- **Cosmos** (FIWARE Cosmos: https://fiware-cosmos.readthedocs.io, accessed on 22 July 2021) provides an interface for integrating Apache Flink and Apache Spark with the rest of the components in the FIWARE Ecosystem. Over the last few years, Apache Flink and Apache Spark have established themselves as the most popular open-source data processing frameworks with the rest of the components of the FIWARE Ecosystem. This GE consists of a set of connectors that allow for receiving data from the Context Broker (through its subscription/notification feature) directly within data processing jobs running in these engines. These connectors allow processing context data in real time, as well as sending the result from this processing back to a given entity in the Context Broker, so other components in the architecture can subscribe to changes in its attributes.

### 5.3. Security Layer

In the FIWARE Ecosystem, several GEs manage authorization and authentication concerning users and devices:

- **Keyrock** (FIWARE Keyrock: https://fiware-idm.readthedocs.io, accessed on 22 July 2021) The Keyrock GE is responsible for Identity Management (**IdM**). Using Keyrock enables OAuth 2.0-based authentication and authorization security to services and applications, as described in [42,43]. In the context of this implementation, Keyrock plays the role of **IdM**: it manages authorization policies (**PAP**) and decides who can access which resources in smart environments.
- **Wilma** (FIWARE Wilma: https://fiware-pep-proxy.readthedocs.io, accessed on 22 July 2021): The Wilma GE brings support of proxy functions within OAuth 2.0-based authentication schemas. It also implements Policy Enforcement Point (**PEP**) functions within an XACML-based access control schema [35]. In the scope of this implementation, several Wilma instances might be needed depending on what service we want to provide or control the access to. Wilma is in charge of enforcing access policies over requests sent to a specific endpoint. When a user or device is authenticated through Keyrock, an OAuth 2.0 token is generated, which must be included in every request sent to any protected component. Wilma intercepts requests and asks Keyrock to validate the token, verifying the identity. Since Keyrock also acts as the Policy Decision Point (**PDP**), it checks the Data Consumer’s (DC’s) access authorization policies. In the case that the request complies with the established policies, Wilma grants access to the requested resource.
- **AuthZForce** (FIWARE AuthZForce: https://authzforce-ce-fiware.readthedocs.io, accessed on 22 July 2021): The AuthZForce GE brings additional support to **PDP/PAP** functions within an access control schema based on the XACML standard.

### 5.4. Application Layer

In this section, we describe the GEs aimed at facilitating the processing, analysis, and visualization of context information for the purpose of implementing the “smart behavior” expected in some of the context-aware systems:

- **Wirecloud** (FIWARE Wirecloud: https://wirecloud.rtfd.io, accessed on 22 July 2021) brings a powerful web mashup platform that eases the development of operational dashboards which are highly customizable by end-users.
- **Kurento** (FIWARE Kurento: https://kurento.rtfd.io, accessed on 22 July 2021) enables real-time processing of media streams supporting the transformation of video cameras into sensors as well as the incorporation of advanced application functions (integrated audiovisual communications, augmented reality, flexible media playing and recording, etc.)
- **FogFlow** (FIWARE Fogflow: https://fogflow.rtfd.io, accessed on 22 July 2021) is a distributed execution framework that supports dynamic processing flows over cloud and edges.

Although FIWARE provides a set of software components that facilitate the visualization and representation of the data, third-party applications can also be easily integrated into this ecosystem by using the NGSI-LD format to represent the data. For example, data generated by a recommendation system can be offered to third parties through an extended Comprehensive Knowledge Archive Network (CKAN) portal enabling the publication of real-time data and the assignment of terms and conditions to data resources or even Complex Event Processing, advanced artificial intelligence or machine learning functions can be implemented on top of the integrated processing engines.

## 6. Example Use Cases

In this section, we present a set of examples to study and validate the proposed reference implementation in different scenarios. First, we present a complete implementation of a Smart Farm scenario that uses big data techniques for building a ruled-based threshold system to trigger automatic actions depending on the inputs received by a set of IoT devices. Then, we provide another example in the Food Industry in which a static dataset of purchases is used for building a machine learning system able to predict the number of purchases on a given date and time.

### 6.1. Smart Farm

In this section, we present an example use case based on the FIWARE step-by-step tutorials (FIWARE Step-by-step tutorial: https://ngsi-ld-tutorials.readthedocs.io/en/latest/iot-sensors.html, accessed on 30 July 2021). This scenario shows a complete digital infrastructure that emulates a Smart Farm.

#### 6.1.1. Data Modeling

All the context data in this scenario are modeled using the Smart Data models defined for Agriculture (Smart Agrifood Domain: https://github.com/smart-data-models/SmartAgrifood, accessed on 30 July 2021). These Data models define a set of mandatory and optional attributes that every entity needs to include. The context of an entity represents the state of a physical or conceptual object which exists in the real world.

**Figure 3:**
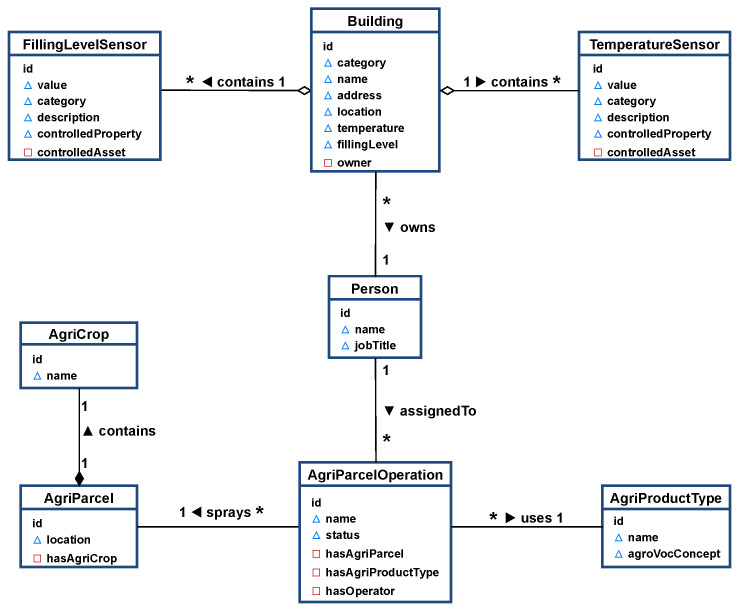
Entities and relationships in the FMIS.

In this scenario, the following set of entities and relationships have been created in order to build a Farm Management Information Systems (FMIS) based on NGSI-LD. For this simplified FMIS, we only need a small number of entities. The relationship between our entities is defined as shown in Figure 3. A **Building** (Building Data Models: https://github.com/smart-data-models/dataModel.Building, accessed on 30 July 2021) is a real-world brick and mortar construct. **Building** entities would have properties such as *name*, *address*, physical *location*, *fillingLevel*, *temperature*, and an association with the *owner* of the building. On the other hand, smart devices such as temperature sensors or filling level sensors would extend a common **Device** data model (Device Data Models: https://github.com/smart-data-models/dataModel.Device, accessed on 30 July 2021). Each **Device** entity would have properties such as: *description*, *category* of device, and association to the asset (*controlledAsset*). A **Person** (Person Data Model: https://smart-data-models.github.io/data-models/common-schema.json, accessed on 30 July 2021) is an entity representing a farmer or farm laborer. Each **Person** entity would have properties such as: *name*, *jobTitle*, and an association to the farm buildings they own (*owner*). Additionally, an agricultural parcel operation or task (**AgriParcelOperation** (Agriculture Parcel Operation Data Model: https://github.com/smart-data-models/dataModel.Agrifood/blob/master/AgriParcelOperation, accessed on 30 July 2021)) is a conceptual entity used to associate workers, agricultural products, and locations. **AgriParcelOperation** entities would have properties such as: *name*, *status* of the task and an association to the worker (i.e., a **Person** entity, *hasOperator*) who performs the task, an association with the product (i.e., an  **AgriProductType** (Agriculture Product Data Model: https://github.com/smart-data-models/dataModel.Agrifood/blob//master/AgriProductType, accessed on 30 July 2021) entity, *hasAgriProductType*) to be used, an association to the parcel (i.e., an **AgriParcel** (Agricultural Parcel Data Model: https://github.com/smart-data-models/dataModel.Agrifood/blob/master/AgriParcel, accessed on 30 July 2021) entity, *hasAgriParcel*) where applied, which points to the crop (i.e., an **AgriCrop** entity (Agricultural Crop Data Model: https://github.com/smart-data-models/dataModel.Agrifood/blob/master/AgriCrop, accessed on 30 July 2021) entity, *hasAgriCrop*) grown.

As can be seen, multiple entities are involved in this scenario. We now present an example of the command for creating the **TemperatureSensor** entity in the Context Broker. In this regard, as shown in Figure 3, the **TemperatureSensor** entity is composed of the attributes: *id*, *type*, *description*, *category*, *controlledProperty*, and *temperature*. This entity can be created in the context broker by the HTTP request presented in Listing 1:

**Figure:**
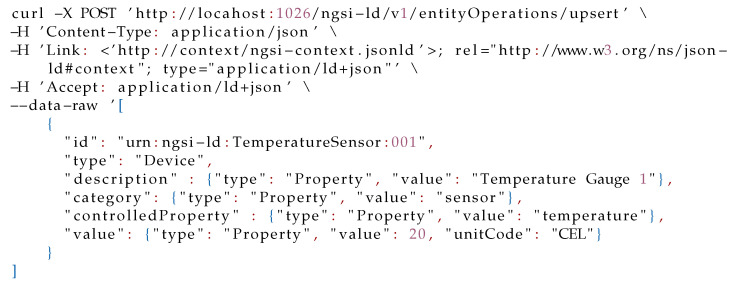
Listing 1: Request for creating a **TemperatureSensor** entity.

#### 6.1.2. IoT Devices

The scenario is composed of a collection of IoT devices. Each one of the devices uses UltraLight 2.0 as a communication protocol on top of HTTP. UltraLight 2.0 is a lightweight text-based protocol for devices and communications where bandwidth and device memory resources are limited [44]. This protocol is structured as a list of key-value pairs divided by the pipe (“|”) character. Below (1) is an example of an UltraLight 2.0 payload containing two attributes “a” with value of “10” and “b” with value “xyz”.

(1) a|10|b|xyz

In this example, the following IoT sensors and actuators are included:

- *Soil Sensor*—It reports the quantity of humidity in the soil.
- *Temperature Sensor*—It measures the ambient or soil temperature at that instant.
- *Filling Sensor*—It shows the feed quantity available in a specific silo.
- *Irrigation System*—It is an actuator that can be activated and open the valves for a small period of time.
- *Animal Collars*—These are devices that allow for showing the current location, the health, and stress level of the animals.
- *Farm Management Information Systems*—These are devices installed in the agricultural machinery that allows communication (send/receive) messages or instructions to laborers, and monitor the current state of the work assigned.

The communication between IoT devices and the Context Broker is achieved by using IoT Agents. These agents define two interfaces for the communication with the Context Broker: southbound and northbound. On the one hand, southbound traffic includes the requests originated by the Context Broker and redirected to an IoT device. Southbound traffic is formed by a set of messages with instructions for the actuator devices in order to change the state of the environment by the execution of those actions. On the other hand, northbound traffic collects the requests originated by the IoT devices and sends them back to the Context Broker. Northbound traffic instead has the measurements gathered by the sensors and collects the state of the environment for including it into context data of the system.

In this regard, the communication schema defined for this example are presented in (2), where the entity id represented in the Context broker is <device_name>, and one of the commands that can be executed is represented by <command> and any other needed values can be added in subsequent parameters <param>.

(2) <device_name>@<command>|<param>|<param>

For example, the message presented in (3) sends a message to a device with the following content: “The Context Broker knows me as ‘urn:ngsi-ld:Robot:001’. I need that the device that is connected to this endpoint execute the command of turn. I have provided the parameters left and 30 (degrees) in order to the device to accomplish the task”.

(3) urn:ngsi−ld:Robot:001@turn|left|30

The corresponding response defined in the northbound interface will be as is stated in (4): “I have complied with a request from the entity known as ‘urn:ngsi-ld:Robot:001’ within the Context Broker. The command I have performed was a turn command. The result was ‘Turnok”’.

(4) urn:ngsi−ld:Robot:001@turn|Turnok

#### 6.1.3. Real-Time Processing and Big Data Analysis

FIWARE relies on microservices for building Smart Solutions. One of the advantages of using this approach is that they are designed to scale up, from basic or simple applications to city-wide systems by means of a huge amount of IoT sensors, actuators, and many other systems like context data providers. As mentioned, any smart solution has as a core context data, and we take advantage of the Context Broker for providing a mechanism that allows us to stay updated about the changes of state and provide notifications as the context changes. In the case of small scenarios, each of the notification events can be treated and processed as it comes by a single endpoint.

Our example implementation in Smart Farming uses the elements and dummy IoT devices described previously. In this regard, the FIWARE components used in this example are chosen for providing a specific task in the whole system. The context broker was chosen as the core for manage all the context data. The IoT Agent for managing the devices that work with the Ultralight 2.0 protocol, and Cosmos Orion Spark Connector for providing the connection between the Context Broker and the Spark Cluster where the program is running. The Spark cluster consists of a single master that acts as a cluster manager to coordinate the execution and some worker nodes to execute the tasks. For providing the historic context information, Orion Context Broker relies on Draco to store the historic in MongoDB.

**Figure 4:**
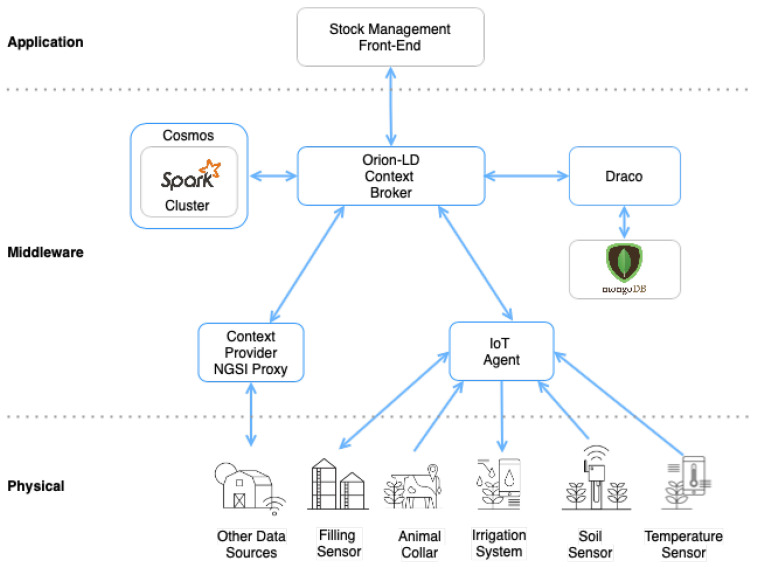
Graphical overview of the smart farming scenario.

Therefore, the overall architecture is presented in Figure 4, and it consists of the following elements:

- Two independent microservices using the FIWARE GEs:
  - The Orion Context Broker for managing the context data by receiving the NGSI-LD requests.
  - The FIWARE IoT Agent for UltraLight 2.0 which will receive southbound requests using NGSI-LD and convert them to UltraLight 2.0 commands for the devices.
- An Apache Spark cluster consisting of a single Cluster Manager and Worker Nodes.
  - The FIWARE Cosmos Orion Spark Connector will be deployed as part of the dataflow which will subscribe to context changes and make operations on them in real time.
- One MongoDB database:
  - Used by the Orion Context Broker to hold context data information such as data entities, subscriptions, and registrations.
  - Used by the IoT Agent to hold device information such as device URLs and Keys.
- This Application does the following:
  - Offers static @context files defining the context entities within the system.
  - Acts as a set of dummy agricultural IoT devices using the UltraLight 2.0.

Once all the components and their connections have been described, we are ready to define the program that will be run over the Spark cluster. In this case, we define a Scala program that provides a rule-based decision application capable of detecting and triggering actions depending on the sensor values. For this specific use case, we have defined a program that opens a water faucet when the soil humidity is too low and turns it back off when the soil humidity is back to normal levels. This way, the soil humidity is always kept at an adequate level. In this regard, Cosmos provides two mechanisms for working with streaming data: one for reading Context data as a Source Operator *NGSILDSource* and other for pushing Context data back to the context broker as a Sink Operator *NGSILDSink*. The dataflow stream uses the *NGSILDSource* operator in order to receive notifications and filters the input to only respond to motion sensors and then uses the *NGSILDSink* to push processed context back to the Context Broker. The code written in scala for building this program is shown in Listing 2. In that program, LOW_THRESHOLD and HIGH_THRESHOLD have been set to 30 and 35, respectively.

**Figure:**
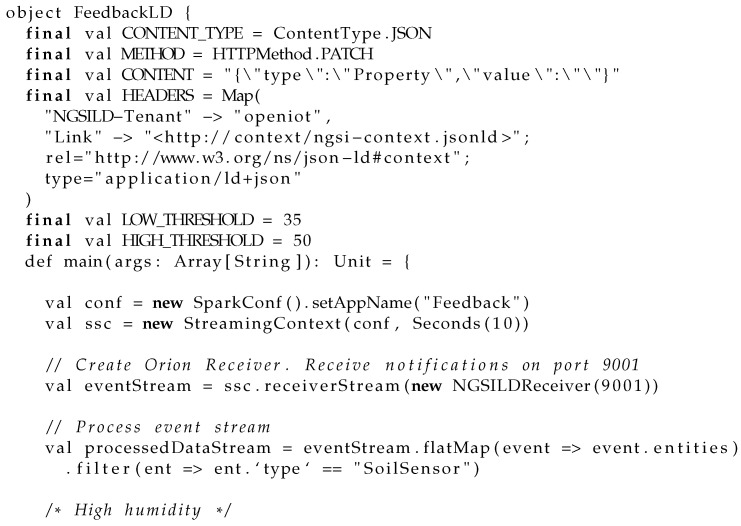

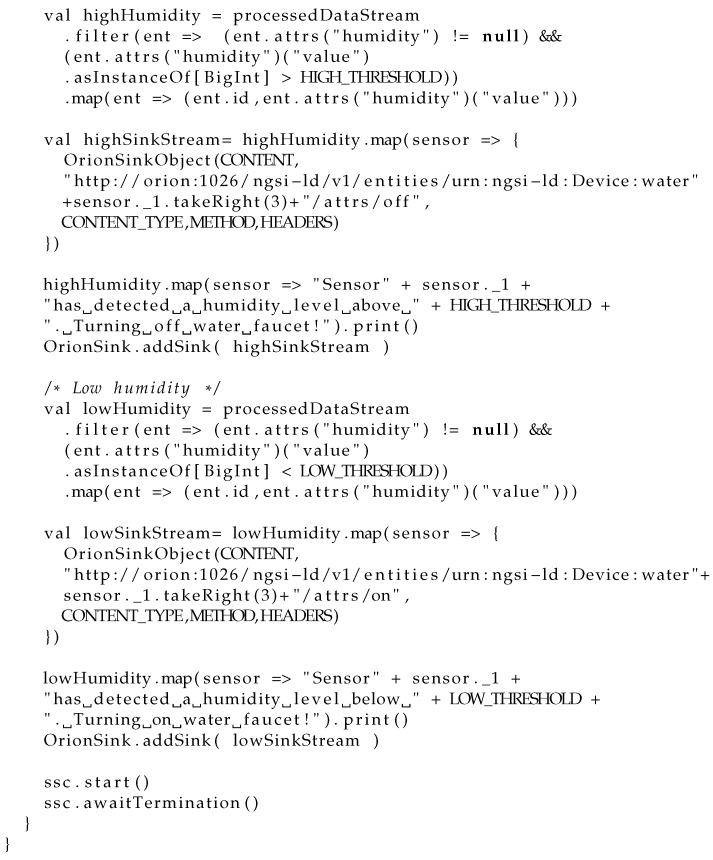
Listing 2: Scala program for controlling humidity level.

The last step for setting up the whole scenario is creating a new subscription as is shown in Listing 3 for listening to the changes of context on the soil humidity sensor so that the Spark program can receive notifications about the changes in the humidity of these entities.

**Figure:**
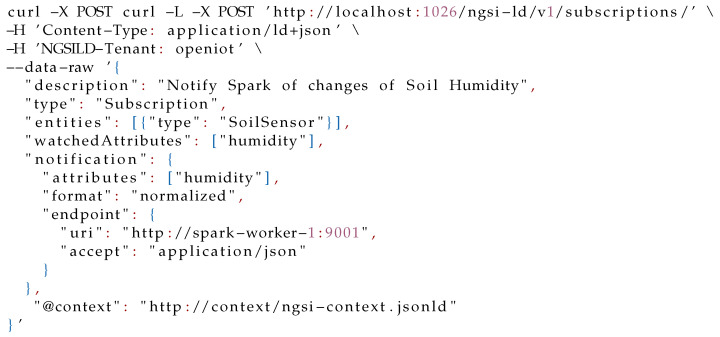
Listing 3: Subscription to the **SoilSensor** entities.

#### 6.1.4. Workflow

**Figure 5:**
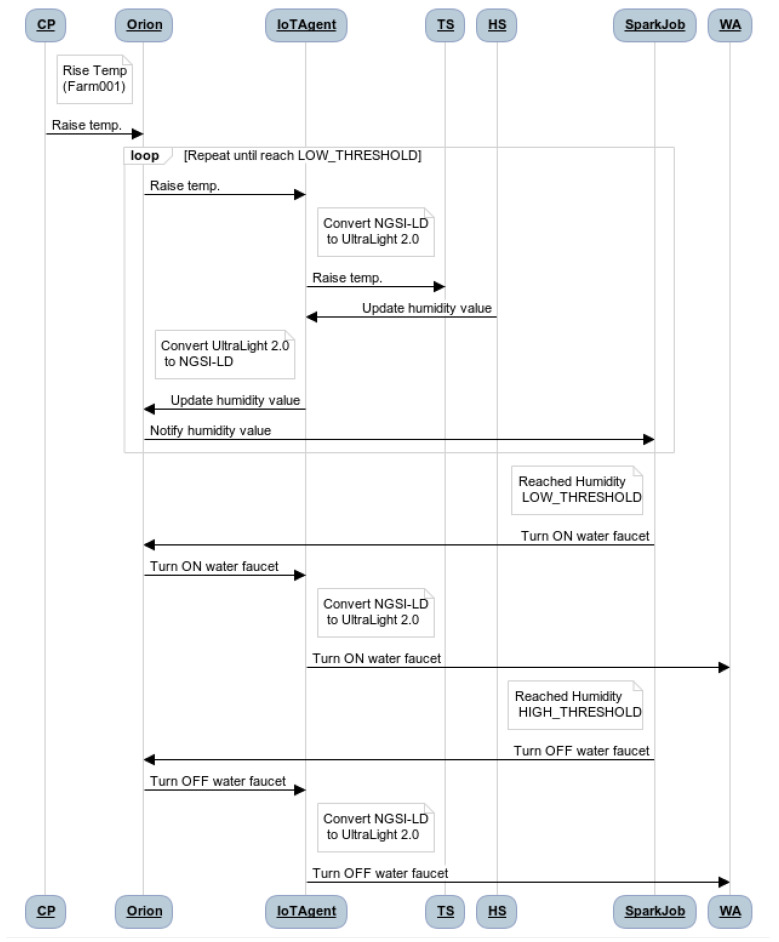
Smart farm scenario workflow.

Once all the services are deployed and entities and subscriptions are created, we can define the workflow of the system presented in Figure 5 and described below.

1. The system begins to generate context data when the user starts some of the devices via the device monitoring interface (Context Provider, CP). For example, the user raises the temperature through Temperature Sensor (TS) in Farm001.
2. The temperature value sent from Orion is in NGSI-LD format. Thus, the IoT Agent (IoTA), takes this value and transforms it into UltraLight 2.0.
3. While the temperature TS in Farm001 is rising, the value of the Humidity Sensor (HS) is decreasing, the value of the HS is sent in a message to Orion via IoTA, converting the messages from UltraLight 2.0 to NGSI-LD beforehand.
4. Orion receives the data generated by the HS and only when it detects that the humidity value is updated, it sends a notification to the Spark Job (SJ) via Cosmos.
5. Since the application is running in the Spark cluster, it is ready for receiving the streaming data from Orion. Thus, when Orion sends a notification, the SJ reads this piece of data from the stream and extracts the humidity attribute value for determining if it is below or under the defined thresholds.
6. Once the SJ detects that the humidity value is under the LOW_THRESHOLD (35), it sends an update for turning on the water faucet in the corresponding water entity hosted in the Orion.
7. When Orion receives the update request from the SJ, it performs the update and sends a notification back to the Water Actuator (WA) via IoTA.
8. IoTA translates to Ultralight the notification containing the update and translates it into a command to the WA and sends it.
9. The WA finally receives the message and turns on the water faucet.
10. This workflow continues until the HS value is above the HIGH_THRESHOLD (50). When this happens, the process is repeated by sending a command to turn off the water faucet to the correspondent device.

### 6.2. Supermarket Purchase Prediction

We present a second example use case in which we use our reference implementation to build a prediction system in the Food Industry. A static dataset of purchases in a grocery store is used for building a machine learning system capable of determining the number of purchases at a given date and time.

**Figure 6:**
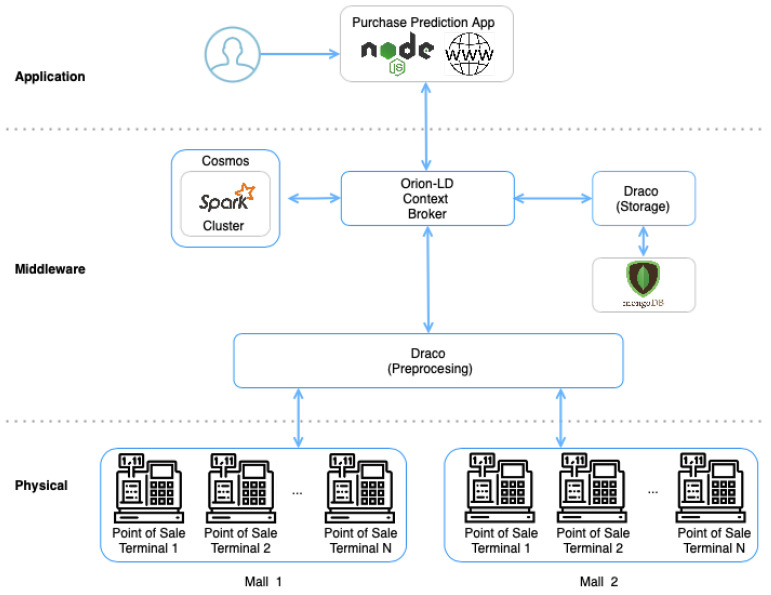
Graphical overview of the Supermarket scenario.

This case presents two independent processes: training the model and deploying the predictor system. First, we use a dataset for building a machine learning model based on the Random Forest Regression Algorithm. This process includes all the stages of the training process such as: data cleaning, feature extraction, algorithm selection, scoring, and tuning. Afterward, the trained model is deployed as a job in a Spark cluster for providing the predictor system. In this stage, we provide an implementation based on FIWARE GEs for providing a complete solution that not only makes predictions but also includes all the context-aware capabilities provided by the Context Broker. A representation of the whole system components is presented in Figure 6

#### 6.2.1. Data Modeling

In this scenario, all data are modeled as **Ticket** entities. However, there does not exist any data model in the FIWARE Smart Data Models initiative for modeling tickets. Consequently, a new data model should be created and published in the Smart Cities domain (Smart Cities Domain: https://github.com/smart-data-models/SmartCities, accessed on 11 August 2021) under a new subject named **Shop**. The first step for creating a new data model is defining its schema. In this model, a **Ticket** entity would have compulsory properties such as: *id* and *type*; optional properties such as: type of ticket (*ticketType*), type of currency *priceCurrency*, total *price*, and date (*dateIssued*); and optional relationships such as products (*hasProducts*). The resulting schema definition is shown in Listing 4, and an example of a **Ticket** entity in Listing 5.

**Figure:**
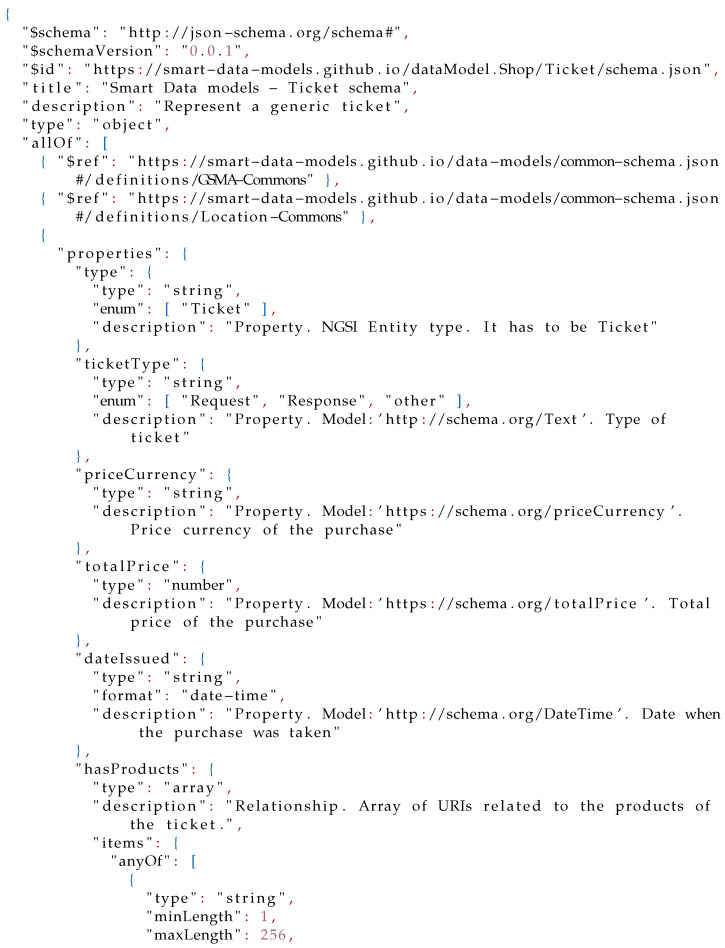

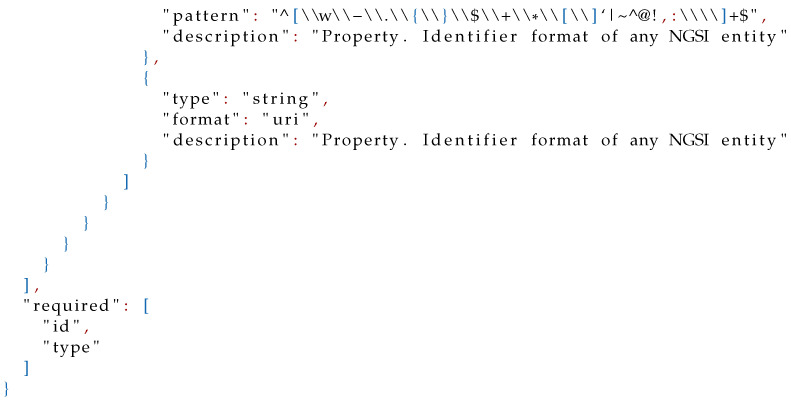
Listing 4: Smart Data Model **Ticket** JSON Schema.

**Figure:**
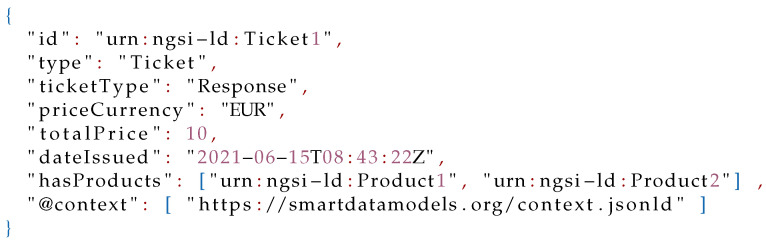
Listing 5: Example of **Ticket** entity.

#### 6.2.2. Data Collection

The data collection process is the first step for building the dataset. A diagram with the representation of this process is shown in Figure 6. In this case, the data are generated by a set of Point of Sale Terminals (POS) located in two different malls. Each of the POS Terminals generates a ticket with the purchase information of each one of the customers that buys a product in that mall. In order to collect these data, each of the terminals sends the ticket to Draco for performing the preprocessing task, i.e., transforming the raw ticket data into an NGSI-LD ticket. Then, the NGSI-LD ticket is sent to the **Ticket** Entity created in the CB. When the CB detects a change in the **Ticket** entity, it sends a notification to the second instance of Draco that is configured to be connected to a MongoDB database for storing the ticket information received by the context broker. Using this data collection pipeline, we can provide an NGSI-LD compliant structured way to store the information of each of the tickets generated in the two stores. Using this approach, we can build a data set with a well-known data structure that can be easily used by any system for further processing.

#### 6.2.3. Model Training

In order to train the model, the first step was to perform data cleaning to avoid erroneous data. Afterward, the feature extraction and data aggregation process were made over the previously described dataset obtaining, as a result, the structure showed in Table 2. In this new dataset, the columns of time, day, month, year, and weekday are set as input and the purchases as the output.

**Figure 7:**
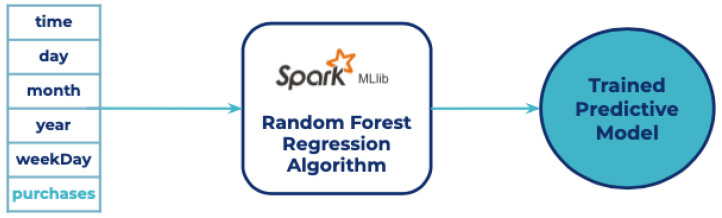
Training pipeline.

The training process was performed using SparkMLlib. The data was split into 80% for training and 20% for testing. According to the data provided, a supervised learning algorithm is the best suited for this case. The algorithm selected for building the model was Random Forest Regression [45] showing a mean square error of 0.22. A graphical representation of this process is shown in Figure 7

#### 6.2.4. Prediction

**Figure 8:**
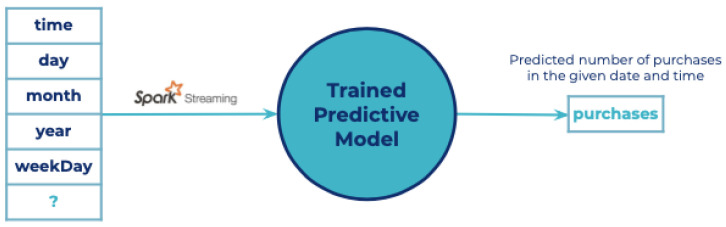
Prediction pipeline.

The prediction system was built using the training model previously defined. In this case, this model is packaged and deployed inside of a Spark cluster. This program uses Spark Streaming and the Cosmos-Orion-Spark-connector for reading the streams of data coming from the context broker. Once the prediction is made, this result is written back to the context broker. A graphical representation of the prediction process is shown in Figure 8.

#### 6.2.5. Purchase Prediction System

**Figure 9:**
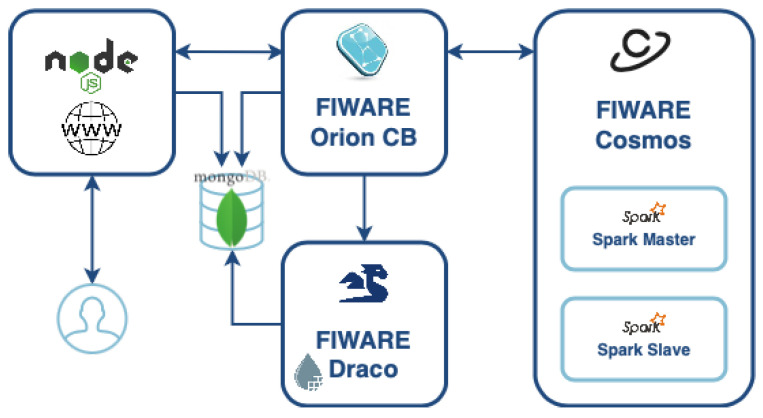
Service components of the purchase prediction system.

**Figure 10:**
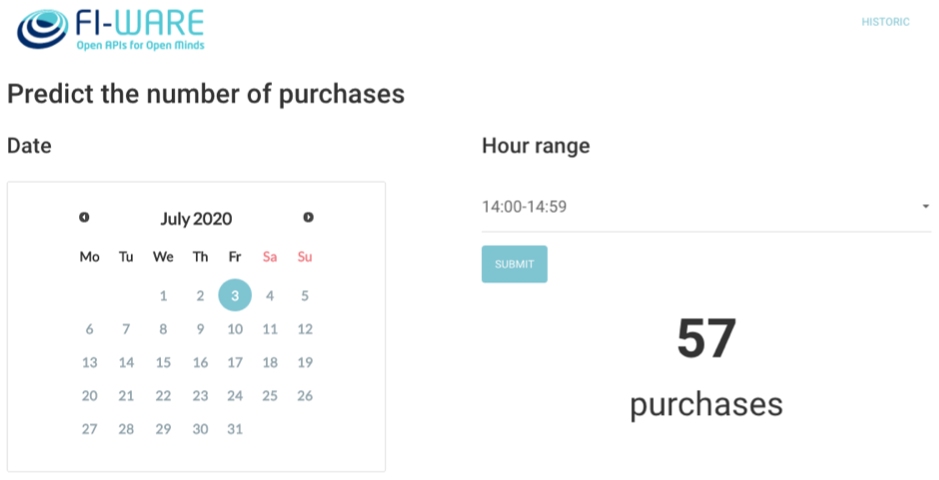
Prediction web application GUI.

In this subsection, we provide an overview of the whole components of the prediction system. The system architecture is presented in Figure 9, where the following components are involved:

- *WWW*—It represents a Node JS application that provides a GUI for allowing the users to make the request predictions choosing the date and time (see Figure 10).
- *Orion*—As the central piece of the architecture. It is in charge of managing the context requests from a web application and the prediction job.
- *Cosmos*—It runs a Spark cluster with one master and one worker with the capacity to scale according to the system needs. It is in this component where the prediction job is running.
- *MongoDB*—It is where the entities and subscriptions of the Context Broker are stored. In addition, it is used to store the historic context data of each entity.
- *Draco*—It is in charge of persisting the historic context of the prediction responses through the notifications sent by Orion.

Two entities have been created in Orion: one for managing the request ticket prediction, *ReqTicketPrediction1*, and another for the response of the prediction *ResTicketPrediction1*.

**Figure 11:**
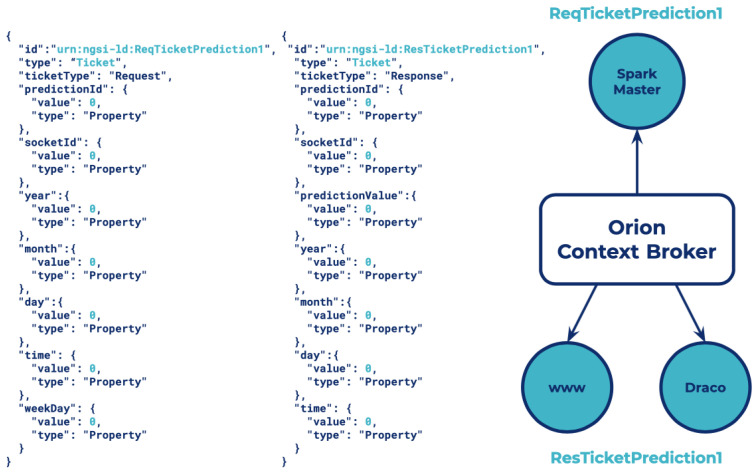
Schema of entities and service subscriptions.

Moreover, three subscriptions have been created: one from the Spark Master to the *ReqTicketPrediction1* entity for receiving the notification with the values sent by the web application to the Spark job and making the prediction, and two more to the *ResTicketPrediction1* entity for notifying the prediction values to Draco for storing the historic context and the web application to present the prediction value to the user. A graphical representation of the component that interacts with the entities, subscriptions, and also the structure of how the two entities with all of their attributes are modeled is presented in Figure 11.

#### 6.2.6. Workflow

**Figure 12:**
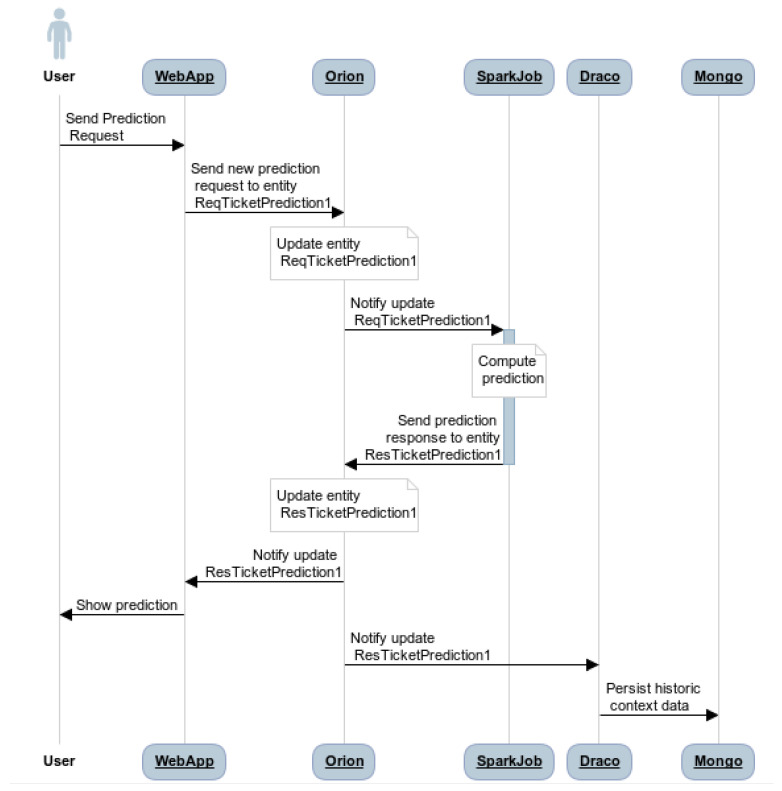
Purchase prediction systems workflow.

The workflow of the system is presented in Figure 12 and is described as follows:

1. The user sends a prediction request to the web application (WebApp).
2. The WebApp generates a new prediction request to the entity. *ReqTicketPrediction1* created in Orion.
3. Orion updates the *ReqTicketPrediction1* entity with the new values and sends a notification with this update to the running SparkJob.
4. SparkJob uses the trained model to compute the prediction based on the values received in the notification.
5. Once the prediction is generated, SparkJob sends a prediction response to the *ResTicketPrediction1* entity stored in Orion.
6. Orion updates the *ResTicketPrediction1* entity and generates two notifications: one for the WebApp and the other for Draco.
7. Once the *ResTicketPrediction1* notification is received by the WebApp, it shows the prediction to the user.
8. Finally, when Draco receives the notification about the update of the *ReqsTicketPrediction1*, it takes the values and persists the historic context data into MongoDB.

## 7. Conclusions and Future Work

This article describes a reference implementation for providing data analytics capabilities to context-aware smart environments. The underlying architecture is divided into four layers, informed by available literature in the field: physical, middleware, application, and security. As such, it considers the complete data lifecycle, from data acquisition through sensors and other IoT devices, to data processing using Big Data technologies and presentation to the end user. Our implementation relies on FIWARE GEs and commonly used open source technologies, a combination that has proven useful in the past for building other types of smart solutions such as digital twins [46], data usage controlled sharing environments [47,48], and enhanced authentication systems [49]. In this article, we show how, by combining these building blocks, we provide a robust, flexible, scalable, and secure way to provide context-aware data analytics in smart environments.

Furthermore, we take advantage of the existing Smart Data Models based on the NGSI standard to easily adapt our reference implementation to different domains. We provide two example use cases to validate the generalizability of our proposal: one in Smart Farming and the other in Smart Industry (retail). These two settings highlight how our reference implementation supports data from a number of different sources, as well as a variety of operations including complex event processing and machine learning. Our hope is that our open-source reference implementation, along with the step-by-step description of our example use cases, offers some clues to researchers, developers, and practitioners on how to operationalize their own context-aware smart environments based on FIWARE.

Further research is needed to determine how the reference implementation presented in this work can be combined with emerging paradigms such as edge computing and fog computing. These new settings bring a set of additional requirements in terms of, e.g., privacy that would require additional investigation, exploring options such as federated learning to implement machine learning capabilities in distributed environments. Another line in need of further investigation is that of providing customized visualization dashboards to end users. Although our reference implementation contemplates the use of the Wirecloud GE for creating web dashboards that allow for easily visualizing the available data, we aim to explore how end users could customize their own data visualization dashboards by adding, e.g., personalized recommendation systems and alarms. For this purpose, we have been working as part of the YODA Action (2019-ES-IA-0121 co-financed by the Connecting Europe Facility of the European Union), aimed at empowering European citizens in the Big Data era.

yes

## Figures and Tables

**Table 1:** Summary of context-aware applications in smart environments.

References	Field	Context Data	Context Processing	Identified Issues	Platform
[13]	Smart Transportation	Time day Day Weather	Naïve Bayesian classifier	Scalability	Ad-hoc
[14]		Dates Location User activities	Context ontology, XML rules	Scalability	Ad-hoc
[15]		User location, Vehicle data, Urban Data	Gradient boosting Decision tree Deep learning	Scalability	Ad-hoc
[16]	Smart Buildings	Temperature Room User location	Rule based threshold	Heterogenous data not addressed	Ad-hoc
[17]		Elevator position User location	Graph based rules	Heterogenous data not addressed Scalability	Ad-hoc
[18]	Smart Farming	Temperature Humidity	Rule Based classifiers	Heterogenous data not addressed Scalability	IBM Bluemix for IoT
[12]		Temperature HumiditySoil Moisture	Rule Based classifiers ML Algorithms	Heterogenous data not addressed Scalability	Cloud-Based
[23,24,25,27]		Temperature Humidity Soil Moisture Weather	Rule Based classifiers ML Algorithms	Outdated standard version	Cloud Based FIWARE

**Table 2:** Sample training dataset.

Time	Day	Month	Year	Weekday	Purchases
6	14	1	2016	3	12
7	14	1	2016	3	12
8	14	1	2016	3	23
9	14	1	2016	3	45
10	14	1	2016	3	55
11	14	1	2016	3	37
12	14	1	2016	3	42
13	14	1	2016	3	41
14	14	1	2016	3	38

## Data Availability

The code and deployment instructions for the Smart Farm use case are available in the following Github repository: https://github.com/FIWARE/tutorials.Big-Data-Spark ( accessed on 19 August 2021) The dataset used in Section 6.2.1 is available at: https://github.com/ging/carrefour_basket_data_challenge ( accessed on 19 August 2021), whereas the repository that contains the whole use case and deployment is in: https://github.com/ging/fiware-ml-supermarket ( accessed on 19 August 2021). Both of these resources provided are available as open source software, and they are free to use under the MIT and Affero General Public License (GPL), respectively. Additionally, a recorded webinar with a video description of the architecture components, their implementation, and a demonstration of the use case presented in Section 6.2 is available at https://www.youtube.com/watch?v=n6XN89VSZNg ( accessed on 30 September 2021).
